# RED: Russian Electoral Data

**DOI:** 10.1038/s41597-026-07590-9

**Published:** 2026-07-22

**Authors:** Konstantin Bogatyrev, Georgy Tarasenko, Nikita Savin

**Affiliations:** 1 Department of Government & Data Science Institute, London School of Economics and Political Science, London, UK; 2 Department of Government, Cornell University, Ithaca, USA; 3 Department of Political Science, University of California, Los Angeles (UCLA), Los Angeles, USA

**Keywords:** Politics, Government

## Abstract

Electoral data at fine-grained administrative levels are essential for analyzing political competition, voter behavior, and electoral institutions. Yet detailed electoral records have been notably restricted, fragmented and inconsistent in many contexts, including contemporary Russia. We introduce a comprehensive, standardized and longitudinally comparable dataset of Russian federal election results at the precinct level, spanning the period 2000–2024. Integrating digital and archival resources, the data include official records on electorate size, turnout, and voting results for all major parties and presidential candidates. The dataset is harmonized across six presidential and five parliamentary election cycles to accommodate frequent precinct renumbering, administrative boundary changes and inconsistencies in reporting. The paper describes the dataset, data sources, and harmonization process, contextualizing them within Russia’s institutional framework and research on authoritarian elections. By offering consistent and harmonized electoral returns, the dataset enables robust empirical analyses of political competition, electoral manipulation, and voter behavior in the period of Russia’s transition to a consolidated autocracy.

## Background & Summary

The availability of detailed election data is essential for robust political analysis. This applies not only to democratic countries but also to non-democratic regimes, where elections consistently raise critical questions about procedural fairness and political pluralism. Contrary to common assumptions, research evidence suggests that elections can have a significant role and influence in all kinds of electoral autocracies, from competitive authoritarian regimes^1,2^ to single-party dictatorships^3–5^. Scholars study elections in authoritarian regimes^6^ to understand how autocrats co-opt elites^7,8^, distribute patronage^9^, respond to popular demands^3–5,10^ and signal strength to both domestic and international audiences^11^, and how voters think and behave under those conditions^2,12–15^. In addition, voting in autocracies and flawed democracies is used for descriptive and methodological research in electoral forensics, i.e., detecting electoral fraud from voting records^16–20^. Given these diverse questions and applications, detailed data are fundamental for empirical research on electoral processes in non-democratic contexts.

This paper introduces the *RED (Russian Electoral Data)* dataset^21^, which comprises the records from all federal elections held in Russia from 2000 to 2024. Transitioning from, at best, a flawed democracy to a personalist autocracy during this period, Russia offers valuable insights into the electoral dynamics and voting behavior in countries experiencing democratic backsliding and the strengthening of authoritarian rule. Indeed, the data span covers all federal elections under Vladimir Putin’s regime, beginning with his first election in 2000. Thus, the data also allow tracking the autocratization of Russia from the early 2000s, when some scholars referred to it as a democracy^22^, to the consolidated authoritarian regime that leaves little doubt among political scientists about its nature^23^. Importantly, despite deepening authoritarianism, Russia held regular presidential and parliamentary elections during this whole period.

Russian elections provide fertile ground for social science research. Political and economic studies based on Russian election data have consistently gained recognition in top academic outlets (see Table 1). However, this research has rarely utilized detailed precinct-level (also known as “polling station-level”) records, let alone for longitudinal analysis. While earlier studies included Russian national aggregates in time-series cross-section samples^24–26^, more recent electoral research on Russia tends to focus on subnational administrative units, such as cities^27–30^, regions^31,32^, or smaller subregional entities^33–36^, as well as analyze subnational elections, such as mayoral^27,30,37^ or regional parliamentary^38^ elections. A few studies on Russia have used precinct-level data, but only cross-sectionally and not over multiple election cycles^20,37,39–41^. Less than a third of 23 studies in Table 1 use panel models that analyze each electoral unit over time, and only two of them use subregional data^28,36^. By providing comprehensive precinct-level electoral returns across multiple election cycles, the RED dataset^21^ addresses these methodological gaps.

**Table 1:** Studies using Russian electoral returns in leading journals.

Study	Panel	Aggregation Level	Nationwide	Time Period	Pres.	Parl.	Subnat.
Remington & Smith 1996, AJPS^61^		Electoral District	*✓*	1993		*✓*	
Moraski & Loewenberg 1999, JOP^24^		National	*✓*	2003		*✓*	
Golosov 2003, CPS^77^		Regional	*✓*	1993–1999		*✓*	
Tavits 2005, AJPS^25^	*✓*	National	*✓*	1995–1999		*✓*	
Alexeev 2006, Polit. Behav.^78^		Regional	*✓*	2003		*✓*	
Pacek *et al*. 2009, JOP^26^	*✓*	National	*✓*	1990–2004	*✓*	*✓*	
Enikolopov *et al*. 2011, AER^33^	*✓*	Electoral District	*✓*	1995–2003		*✓*	
Goodnow & Moser 2012, CPS^34^		Subregional		1995		*✓*	
Enikolopov *et al*. 2013, PNAS^20^		Polling Station		2011		*✓*	
Lazarev *et al*. 2014, WP^39^		Polling Station		2007–2008	*✓*	*✓*	
Buzin *et al*. 2016, Elec. Stud.^40^		Polling Station		2011		*✓*	
Reuter *et al*. 2016, CPS^27^		City	*✓*	2001–2012			*✓*
Rundlett & Svolik 2016, APSR^41^		Polling Station	*✓*	2011–2012	*✓*	*✓*	
Beazer & Reuter 2019, JOP^28^	*✓*	City	*✓*	2003–2012		*✓*	
Belmonte & Rochlitz 2019, JEBO^31^	*✓*	Regional	*✓*	1999–2012	*✓*	*✓*	
Enikolopov *et al*. 2020, Econometrica^29^		City	*✓*	1995–2003		*✓*	
Buckley *et al*. 2022, CPS^30^		City	*✓*	2002–2018			*✓*
Gandhi *et al*. 2022, Elec. Stud.^35^		Electoral District	*✓*	2003		*✓*	
Minaeva *et al*. 2023, Elec. Stud.^36^	*✓*	Subregional	*✓*	2008–2018	*✓*		
Szakonyi 2023, AJPS^38^		Subregional	*✓*	2009–2017			*✓*
Borisova *et al*. 2024, APSR^37^		Polling Station		2010s			*✓*
Teplyshova 2026, Elec. Stud.^32^	*✓*	Regional	*✓*	2000–2024	*✓*		
Enikolopov *et al*., Forthcoming, EJ^79^		Polling Station		2011–2016		*✓*	

*Note:* This table surveys general interest or social science journals that use electoral returns from Russia, excluding Soviet and pre-revolutionary periods. It does not cover area studies and electoral forensics, where Russian voting data have been extensively used^19,80^. Notation: *✓* = includes feature; blank = not used. Panel = electoral panel data; Pres. = presidential elections; Parl. = parliamentary elections; Subnat. = subnational elections.

Although using restricted samples and less granular data can sometimes be justified by case selection^20,34,37,39^, it raises the question of how many studies have either been abandoned, substantially altered, or shifted towards survey data collection. Providing clean and standardized electoral returns can help expand the methodological toolkit for studying Russian politics, especially given the growing challenges of survey research in repressive environments^42–44^. Table 1 shows that none of the reviewed top-journal studies simultaneously analyzes (1) countrywide (2) precinct-level electoral returns (3) with repeated observations of the same polling stations over time. The RED dataset^21^ meets these criteria, providing nationwide precinct-level data for presidential (2000, 2004, 2008, 2012, 2018, 2024) and parliamentary (2003, 2007, 2011, 2016, 2021) elections.

Our dataset contributes to recent scholarly efforts to systematically collect and standardize electoral data at subnational geographic scales, joining comparable initiatives focused on the United States^45–47^, India^48,49^, Indonesia^50^, Mexico^51^ and across Europe^52–57^. Such harmonized datasets facilitate comparative research into electoral behavior, institutional dynamics, and political geography, allowing researchers to explore cross-national patterns and local determinants of political outcomes and representation^58^. By consolidating and harmonizing election data across years, regions, and polling stations, the RED dataset^21^ provides a critical tool for empirical research on Russian elections and electoral authoritarianism.

## Methods

### Data Collection and Coverage

Electoral results in Russia have been notably public, especially compared to other authoritarian regimes (for example, neighboring Belarus typically publishes only aggregated results at the regional level, without precinct-level disclosure). This relative openness is largely due to an automated system for storing voting records commissioned in the mid-1990s and officially launched in 2000. However, despite making individual precinct-level results viewable online, the Central Election Commission (CEC) of Russia never provided complete electoral datasets in downloadable formats. As a result, researchers, activists and enthusiasts had to scrape these data page-by-page, a task that has become increasingly difficult in recent years due to CAPTCHA-based restrictions and periodic reformatting of the Commission’s website (http://izbirkom.ru, inaccessible from outside Russia). The data systematically collected and redistributed by activists and researchers are stored in various repositories, which we also used as data sources^59,60^. While these data have proven immensely valuable to researchers and the broader public, without further processing the data downloaded from the CEC’s website contained notable missing points and remained inconsistent and unharmonized across election cycles.

The RED dataset^21^ aims to provide a significant advancement over existing collections by integrating and harmonizing precinct-level electoral returns across all federal election cycles since 2000 within the internationally recognized territory of the Russian Federation (see Table 2). We begin the series in 2000 as earlier elections have either fully absent^61^ or highly incomplete precinct-level coverage in published official sources. Alongside the precinct-level data from traditional offline voting, the dataset also covers the recently introduced online voting, reported at the electoral district level for the 2021 parliamentary elections and at the regional level for the 2024 presidential elections, for those regions where online voting was implemented.

**Table 2:** Dataset coverage.

Year	Election Type	# Regions	# TIKs	# UIKs
2000	Presidential	82	2,593	92,124
2003	Parliamentary	83	2,682	95,197
2004	Presidential	83	2,684	95,426
2007	Parliamentary	83	2,688	95,835
2008	Presidential	83	2,688	96,248
2011	Parliamentary	83	2,688	94,849
2012	Presidential	83	2,688	95,030
2016	Parliamentary	83	2,687	95,466
2018	Presidential	83	2,687	95,911
2021	Parliamentary	83	2,693	94,929
2024	Presidential	83	2,714	90,308
Overall		912	29,492	1,041,323

Table 3 provides an overview of the sources used to compile the electoral dataset. The primary source of raw data are precinct-level election returns scraped from the Central Electoral Commission (CEC) website by Sergey Shpilkin (for 2000–2021) and Ivan Shukshin (for 2024), which we accessed via Dmitry Kobak’s public GitHub repository^59^ https://github.com/dkobak/elections/tree/master/data. These datasets include detailed information on precinct identifiers, electorate, turnout, and vote counts, and provide a foundation for cross-sectional analyses of electoral outcomes within electoral cycles. While official electoral statistics are generally comprehensive, a few regions exhibited missing or incomplete precinct-level data, most notably the Chechen Republic and the Republic of Sakha (Yakutiya) in 2000. To address this gap, we manually included the 2000 electoral records from a printed volume^62^ that we obtained from the National Library of the Republic of Sakha (Yakutia) and digitized. For purposes of polling station geocoding and data harmonization, we further sourced the data on polling station addresses downloaded by activists from the CEC’s website. In particular, the 2012 raw address data were collected from the GIS-Lab project website^63^ https://gis-lab.info/qa/webvybory2012.html,while the addresses in subsequent years are available on Ivan Shukshin’s “Commissions of the Russian Federation” portal^60^ https://commissions.notelections.ru.

**Table 3:** Data sources used in constructing RED.

Data Type	Years	Source
Election returns (excl. Yakutia 2000)	2000–2021	Data scraped by Sergey Shpilkin from the Central Electoral Commission of Russia^59^
Yakutia 2000 election returns	2000	*Electoral Handbook*, volume published by the Central Electoral Commission of Sakha (Yakutia)^62^
Election returns	2024	Data scraped by Ivan Shukshin from the Central Electoral Commission of Russia^59^
Polling station addresses	2012	Data scraped by GIS-Lab activists from the government website webvybory2012.ru^63^
Polling station addresses	2016–2024	‘Electoral Commissions’ Database scraped by Ivan Shukshin from the Central Electoral Commission of Russia^60^

### Data Cleaning and Preprocessing

The raw electoral returns were extensively cleaned and processed to create a coherent and analytically consistent dataset. Original data from different election cycles exhibited significant variations in structure, format, and naming conventions, requiring extensive harmonization and standardization. The processing was done in R, and the code is provided in the replication package (https://github.com/georgytarasenko/RED-replication-package).

First, we extracted and consistently structured the electronic records from each election cycle to ensure comparability over time. These include region, electoral district (OIK), territorial electoral commission (TIK), precinct (UIK), electorate size, turnout by voting mode (early, offsite, onsite), invalid and valid votes, and votes cast for major political parties and candidates. Because the original datasets varied substantially in both column names and formats, we programmatically identified, renamed, and aligned these fields to ensure comparability across years.

Second, we integrated the digitized electoral results from Sakha (Yakutia) in 2000 into the cleaned dataset, enhancing the completeness of the data coverage.

Third, we explicitly excluded territories outside of Russia’s internationally recognized borders.

Fourth, we processed online voting results separately due to their distinct aggregation levels and inconsistent reporting in the raw data. For regions where and years when online voting was implemented, we reclassified online votes initially recorded as onsite turnout into a dedicated online turnout variable and flagged them accordingly in the municipal commission field. Consistent with the aggregation levels available from the official electoral returns, the online voting data are at the electoral district level for 2021 and at the regional level for 2024, without precinct identifiers as these do not correspond to any actual polling stations.

Finally, we addressed substantial discrepancies in geographic naming conventions. The raw data contained frequent inconsistencies in the naming of SMD and territorial electoral commissions (TIKs), such as differences in spelling, the use of Latin versus Cyrillic characters, and inconsistent administrative prefixes and suffixes (e.g., numbers, “urban” or “rural” qualifiers). We standardized these names through systematic text processing using regular expressions, ensuring uniformity across election cycles. This involved removing numerical prefixes, translating Latin letter incursions to their Cyrillic equivalents, removing auxiliary administrative terms, and harmonizing grammatical forms of place names.

A similar procedure was conducted with precinct addresses. We preprocessed highly incongruent addresses retrieved from open sources^60,63^ using Llama-3.1-8B-Instant^64^ via the Groq API. Llama-3.1-8B-Instant is an open-weights model and can also be accessed through alternative API providers or run locally. The model was prompted to extract seven standardized fields (municipality, locality type, locality name, street, house number, building name, and postal code) into structured JSON format. The complete prompt template was:

Please extract and standardize the following Russian address into a valid JSON object with the following fields: municipality, locality type, locality name, street, house number, building name, postal code. Return a clean JSON object only. Use 'NA' where uncertain. Expand abbreviations and use Cyrillic script only.

To ensure reproducibility, we set the temperature parameter to 0 in all API calls (the temperature parameter controls output randomness; temperature = 0 makes the model deterministic by always selecting the highest-probability token).

Once this initial cleaning was complete and a uniform structure and naming convention established, we were able to proceed with a systematic longitudinal harmonization of electoral units across cycles, which we describe in the next section.

### Data Harmonization

Precinct electoral commissions (UIKs) are the lowest-level electoral units in Russia, nested within territorial electoral commissions (TIKs), which usually correspond to municipalities and are themselves nested within federal subjects, i.e. regions. Each region is further divided into single-member districts (SMDs) for parliamentary elections under the mixed-member system. Over time, regional, subregional and precinct identifiers may change due to renaming, administrative reorganizations, or renumbering. Our harmonization procedures address these challenges across levels to enable longitudinal analysis of electoral returns from 2000 to 2024, aiming to align the data structure with its 2024 state.

#### Harmonizing regions, electoral districts and territorial commissions

Prior to precinct-level harmonization, we standardize higher-level electoral units (regions, SMDs and territorial commissions) to ensure continuity across election cycles. This includes standardizing geographic naming conventions of the same units over the years as well as retrospective border changes. Names of regions, SMDs and TIKs are textual and prone to inconsistency across elections, due to either administrative changes (e.g., redistricting, splits and mergers), renaming or variations of spelling.

We address renaming and spelling inconsistencies by applying a standardized name retrospectively to the same unit while preserving historical names of entities in a separate variable. Regional and electoral district border changes are addressed by reassigning the respective TIKs currently belonging to that region or district to it retrospectively in earlier years. Mergers and splits are handled by maintaining a stable entity across all election cycles. For mergers, we retrospectively combine units in the pre-merger years. For splits, we retrospectively apply the post-split identifiers to earlier years where feasible, and we prospectively re-merge the entity in the post-split years otherwise. Additionally, for roughly 30 cases where territory moved between two TIKs that both continued to exist under unchanged names, we expand the matching search space to include the partner TIK identified through context information. Such territorial transfers are rare, as most TIKs correspond to municipalities, which typically change their borders through splits and mergers.

#### Precinct continuity diagnostics

Unlike TIKs, precincts have no names and are identified solely by numeric codes. These precinct numbers are assigned independently within each TIK and region and are periodically renumbered or reassigned over time. Consequently, the same numeric code may refer to different precincts in different elections, even within the same TIK. This poses serious challenges in tracking electoral behavior at the precinct level over time.

To understand the structure of precinct continuity, we perform descriptive diagnostics prior to harmonization. Of the 121,684 unique region-precinct combinations observed between 2000 and 2024, only 71,142 are present in all the elections under analysis. However, presence alone does not guarantee continuity: precinct numbers may be reused for different precincts after renumbering. Therefore, we mark all cases where many precincts disappear and are replaced with new numbers between elections, indicating likely reassignments of identifiers without corresponding boundary changes.

For these flagged precincts, we harmonize precinct identifiers across elections from 2000 to 2024. Our harmonization procedures combine sequence alignment for earlier years with address-based techniques in later years. Our approach relies on polling station matching, which primarily resolves renumbering but cannot capture changes in precinct boundaries. As a result, longitudinal comparisons at the precinct level may introduce measurement error where boundaries might have changed.

#### Pre-2012: Dynamic programming alignment within TIKs

Between 2000 and 2011, polling station address data are not consistently available. For flagged precincts, we implement a sequence alignment algorithm using dynamic programming (DP) to match polling stations from one election year to the next (e.g., from 2000 to 2003, 2003 to 2004, etc.). This algorithm globally minimizes a cost function across all precincts within a TIK while preserving the order of precinct numbers. In contrast to greedy approaches—which match one precinct at a time and are prone to cascading mismatches—DP ensures optimal global alignment even in the presence of significant renumbering.

Let ${U}^{(t)}=({u}_{1}^{(t)},\ldots ,{u}_{m}^{(t)})$ and ${U}^{(t+1)}=({u}_{1}^{(t+1)},\ldots ,{u}_{n}^{(t+1)})$ denote the ordered sequences of precincts in a given TIK in two consecutive elections. Each precinct is associated with an electorate size, denoted ${e}_{i}^{(t)}$ or ${e}_{j}^{(t+1)}$. We define a dynamic programming table *D*(*i*, *j*) to represent the minimum cumulative cost of aligning the first *i* elements of *U*^(*t*)^ with the first *j* elements of *U*^(*t*+1)^ under the following recurrence:

$$D(i,j)=\min \left\{\begin{array}{ll}D(i-1,j-1)+{c}_{{\rm{match}}}(i,j) & \,{\rm{(match)}}\\ D(i-1,j)+{c}_{{\rm{skip}}} & \,{\rm{(skip\; old)}}\\ D(i,j-1)+{c}_{{\rm{skip}}} & \,{\rm{(skip\; new)}}\end{array}\right.$$

where:

- $${c}_{{\rm{match}}}(i,j)=\left\{\begin{array}{ll}| {e}_{i}^{(t)}-{e}_{j}^{(t+1)}| , & \,{\rm{if}}\,| {e}_{i}^{(t)}-{e}_{j}^{(t+1)}| \le \delta \\ \infty , & \,{\rm{otherwise}}\end{array}\right.$$ is the cost of matching two precincts based on electorate difference, with *δ* (typically 500) as the tolerance threshold.
- *c*_skip_ is a constant penalty for skipping a precinct in either sequence (e.g., 200).

The algorithm reconstructs the globally optimal alignment by tracing back through the DP matrix. This method allows us to generate 1:1 precinct correspondences across elections where renumbering occurred, provided that the underlying precincts remained geographically stable. It also permits skipping unmatched precincts, accommodating real-world cases where precincts are added or removed.

Matched precincts are assigned consistent harmonized identifiers. The original precinct codes are preserved in a separate column uik_old, ensuring transparency. Unmatched precincts are generally left unchanged; however, if their identifier is reassigned to another precinct via matching, they are retrospectively renumbered to avoid duplication. All conflicting identifiers are resolved using globally unique values generated from a counter that increments across transitions.

#### Post-2012: Fuzzy address-based matching

From 2012 onward, official data include precinct-level address strings. These allow for more reliable harmonization across elections, even in the presence of renumbering, splits, or mergers. We implement a fuzzy matching algorithm that compares cleaned address strings within the same TIK across elections, using the Jaro-Winkler string distance to identify likely matches, with a maximum distance threshold of 0.05.

We restrict matching to precincts within the same TIK and require 1:1 matches. When multiple polling stations tie at the minimum address distance, we select the one with the most similar electorate size, since genuinely-same precincts tend to exhibit limited electorate drift across elections. Where matching yields high-confidence pairs, we assign consistent harmonized identifiers. The original precinct identifiers are preserved in a separate uik_old column across all years, allowing full traceability.

As in the earlier period, any unmatched precinct whose original identifier conflicts with a newly assigned value is also renumbered. These conflicts are resolved using a global counter to ensure uniqueness across all years and regions. After harmonization, we obtain 195,366 unique precincts from an initial pool of 1,041,323 precinct-election observations (see Table 4). Of these, 31,237 are present in all eleven elections in the dataset, representing about one-third of the 90,000-95,000 precincts active in any given cycle. Across the four post-2013 cycles, 78,586 precincts are continuously observed, reflecting both the greater stability of precinct identity after the 2013 introduction of five-year terms for polling station workers as well as the availability of consistent polling station address data in these years.

**Table 4:** Top-10 panel presence patterns in the harmonized dataset, restricted to precincts observed in at least two election years.

	’00	’03	’04	’07	’08	’11	’12	’16	’18	’21	’24	Count
1	*✓*	*✓*	*✓*	*✓*	*✓*	*✓*	*✓*	*✓*	*✓*	*✓*	*✓*	31,237
2								*✓*	*✓*	*✓*	*✓*	29,144
3	*✓*	*✓*	*✓*	*✓*	*✓*	*✓*	*✓*					23,625
4	*✓*	*✓*	*✓*									9,957
5	*✓*	*✓*	*✓*	*✓*	*✓*							7,207
6				*✓*	*✓*	*✓*	*✓*	*✓*	*✓*	*✓*	*✓*	5,400
7						*✓*	*✓*	*✓*	*✓*	*✓*	*✓*	5,093
8						*✓*	*✓*					4,455
9								*✓*	*✓*	*✓*		4,371
10		*✓*	*✓*	*✓*	*✓*	*✓*	*✓*	*✓*	*✓*	*✓*	*✓*	4,333
**Total Unique Precincts in Dataset:**												**195,366**

Top-10 Patterns Shown: 124,822 precincts. Total Unique Precincts in Dataset: 195,366.

## Data Records

The final harmonized dataset is available at Harvard Dataverse^21^ in two formats: red.Rds (native R format) and red.csv (comma-separated values). The replication package — including raw data, intermediate files, and all analysis scripts — is available on GitHub (https://github.com/georgytarasenko/RED-replication-package). Each row of the dataset corresponds to one polling station (UIK) in one election year. Variables fall into five groups. Election identifiers capture the year and type of election (presidential or parliamentary). Geographic and administrative identifiers record the federal subject, the harmonized and original territorial commission names (anchored to 2024 boundaries), and — for parliamentary elections — the single-member district codes; geocoded latitude and longitude are available for 2012–2024. Precinct identifiers provide both the harmonized UIK number (indexed to 2024 boundaries) and the original precinct number as reported in the raw CEC data for each election year. Turnout variables include the total electorate, votes cast, valid and invalid ballots, turnout rate, and disaggregated counts for onsite, offsite (mobile), early, absentee, and electronic voting; the electronic voting variable is non-zero only in aggregated online-voting rows appended to the precinct-level data and should not be pooled with regular precinct rows in turnout analyses. Finally, vote variables record raw vote counts and, for major candidates and parties, vote shares, covering all presidential candidates and party lists across the eleven election cycles in the dataset.

## Data Overview

### Context

The RED dataset^21^ includes detailed information on election results, voter turnout, and precinct-level dynamics, which are essential for examining trends in electoral behavior, manipulation, and the broader implications for political stability.

The RED dataset^21^ includes records from Russian presidential and legislative elections covering the period from 2000 to 2024. This timeframe begins with the onset of Vladimir Putin’s presidency and encompasses key institutional changes in the country’s electoral system. Russian presidential elections were held every four years from 2000 to 2012 and subsequently every six years following a constitutional reform that extended the presidential terms. They use a two-round runoff system in a single national constituency, requiring an absolute majority to declare victory in the first round – a condition always fulfilled throughout the observed period, resulting in no second rounds.

Similarly, the legislative elections of the State Duma (the lower chamber of the Russian parliament) were held every four years until 2011, and every five years thereafter due to a constitutional reform which extended legislative terms. During this period, Russia’s parliamentary election system was repeatedly restructured, exemplifying one of the key strategies of authoritarian control in electoral regimes^65^. It alternated between a mixed-member majoritarian-proportional system and a closed-list proportional representation system during the observation period^66^. Until 2003, 225 members of parliament referred to as deputies were elected from single-member districts (SMDs) using a plurality rule, and 225 more were elected through closed party lists with proportional representation (PR) and a 5% national threshold. For the 2007 and 2011 elections, the system was changed to pure PR with a 7% threshold and a ban on electoral blocs. Scholars argue that this reform was designed to impose discipline on legislators through centralized control over nominations^35^. The mixed system was later reinstated, reintroducing 225 SMDs and lowering the threshold for PR representation back to 5%, effective from the 2016 election cycle. The dataset reports votes for party lists (i.e., the PR vote), which provides the most consistent basis for longitudinal analysis across parliamentary elections.

A notable institutional innovation in recent election cycles has been the introduction of online voting, officially referred to as remote electronic voting (as opposed to onsite electronic voting machines). At the federal level, online voting has gradually expanded, starting with two regions for the 2020 constitutional referendum, increasing to six for the 2021 parliamentary elections and already involving 27 out of 83 regions covered by our dataset by 2024^32,66^. In these regions, online voting operated in parallel to traditional offline voting and accounted for over 2.5 million votes in 2021 and over 8 million votes in 2024. While the government presented it as convenience, available evidence has documented workplace coercion to vote online^67^ and highlighted reduced integrity of the online voting compared to its offline alternative^68,69^.

**Fig. 1:**
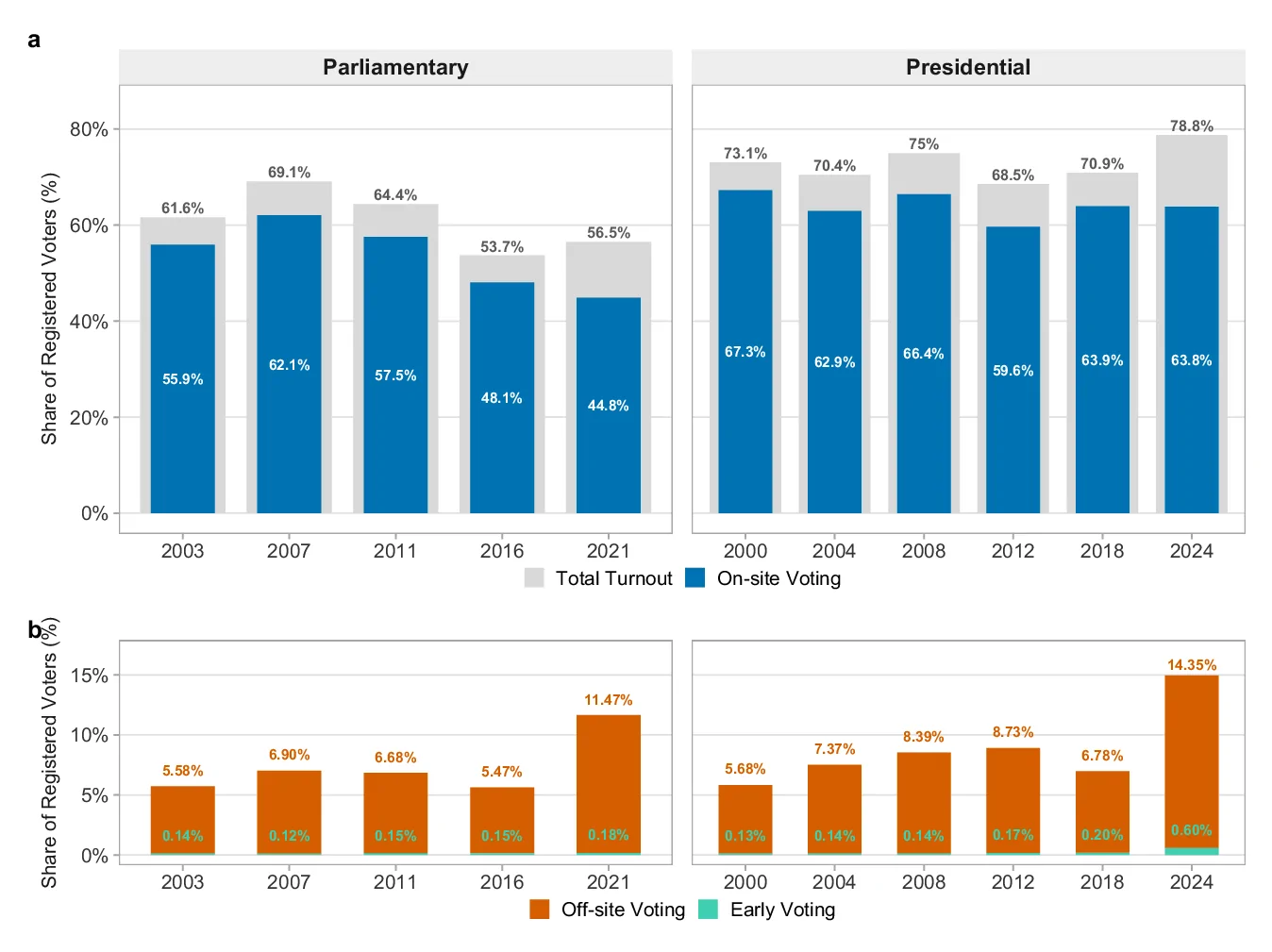
Turnout composition across election years: on-site, off-site and early voting.

Figure 1 illustrates the composition of voting modes in Russian parliamentary and presidential elections from 2000 to 2024. The top panels compare overall voter turnout (light gray) with on-site voting (dark gray). Presidential elections generally exhibit higher overall turnout and on-site participation than parliamentary elections. The bottom panels break down alternative voting modes into early voting (blue) and off-site voting (pink). Off-site voting constitutes the majority of non-on-site votes and has grown steadily over time, reaching 14.3% in the 2024 presidential election. Early voting, while limited in scale, shows a significant upward trend: for example, the share of this mode grew 2.2 times in the presidential elections between 2000 and 2024.

### Electoral Geography

**Fig. 2:**
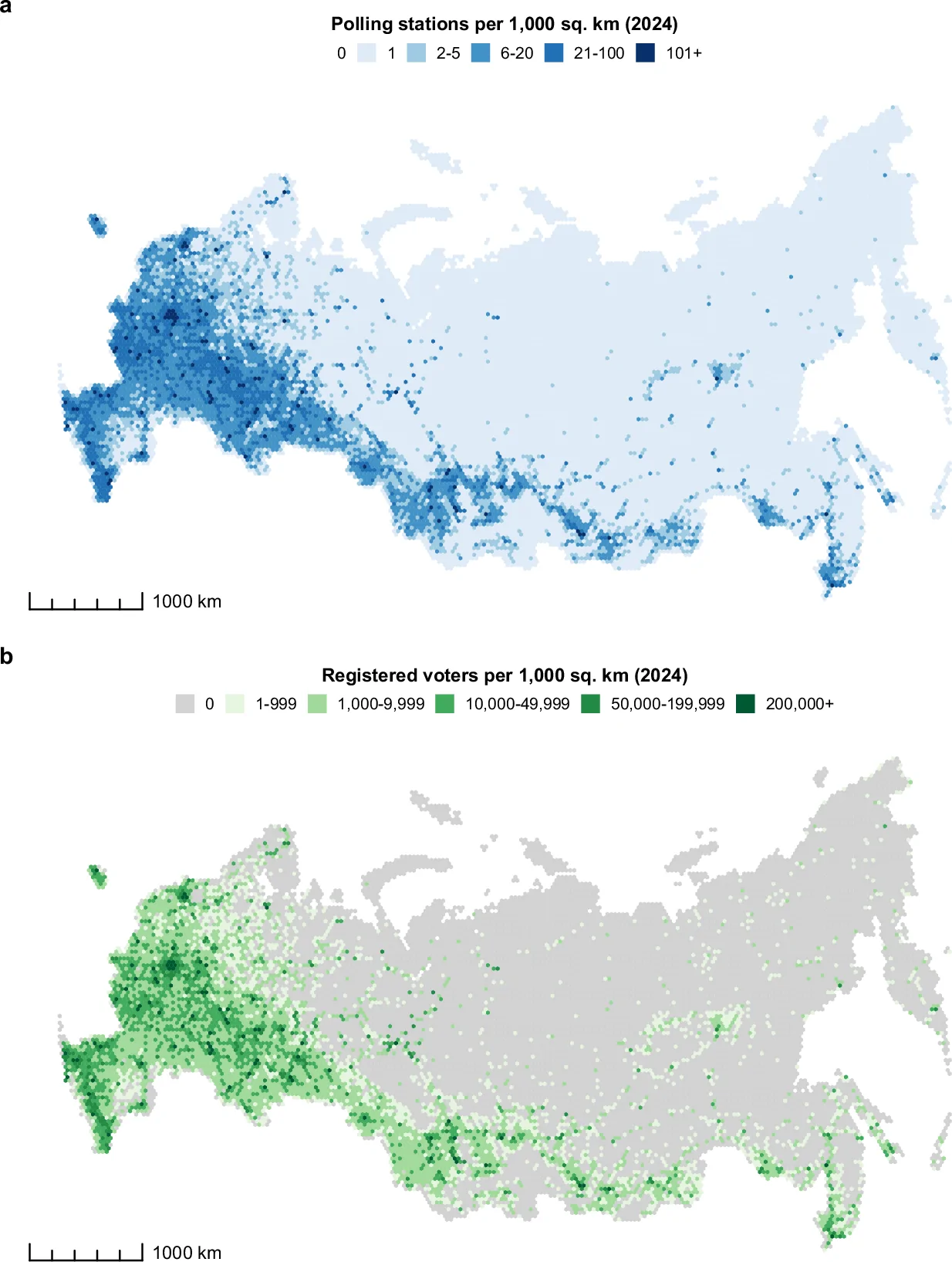
Spatial distribution of electoral infrastructure and voters.

Russia’s vast geography has long shaped its electoral system. Figure 2 displays the latest data on the spatial distribution of polling station infrastructure and registered voters across Russia in 2024, using a uniform 1,000 *km*^2^ hexagonal grid. Panel (a) shows the density of polling stations, while Panel (b) presents the density of the registered electorate. Largely consistent with the spatial distribution of the population, the maps show clustering of both infrastructure and population in the western part of the country and major urban centers, with sparse coverage across much of Siberia and the Far East. The only notable exception is the sparsity of data in the Belgorod region, where elections were suspended in many areas due to shelling by the Ukrainian military.

The regional dimension is essential to understand the electoral dynamics in Russia. As a federal state by constitutional design, Russia has historically exhibited substantial subnational variation, particularly in the early 2000s, when electoral outcomes across regions reflected divergent economic structures, local elites, and degrees of autonomy^34,70,71^. While the consolidation of authoritarian rule has since reduced visible regional differentiation, territorial dynamics continue to matter: they shape how central control is asserted, where resistance or divergence emerges, and how regime support is manufactured and maintained across Russia’s vast and heterogeneous landscape^72,73^.

**Fig. 3:**
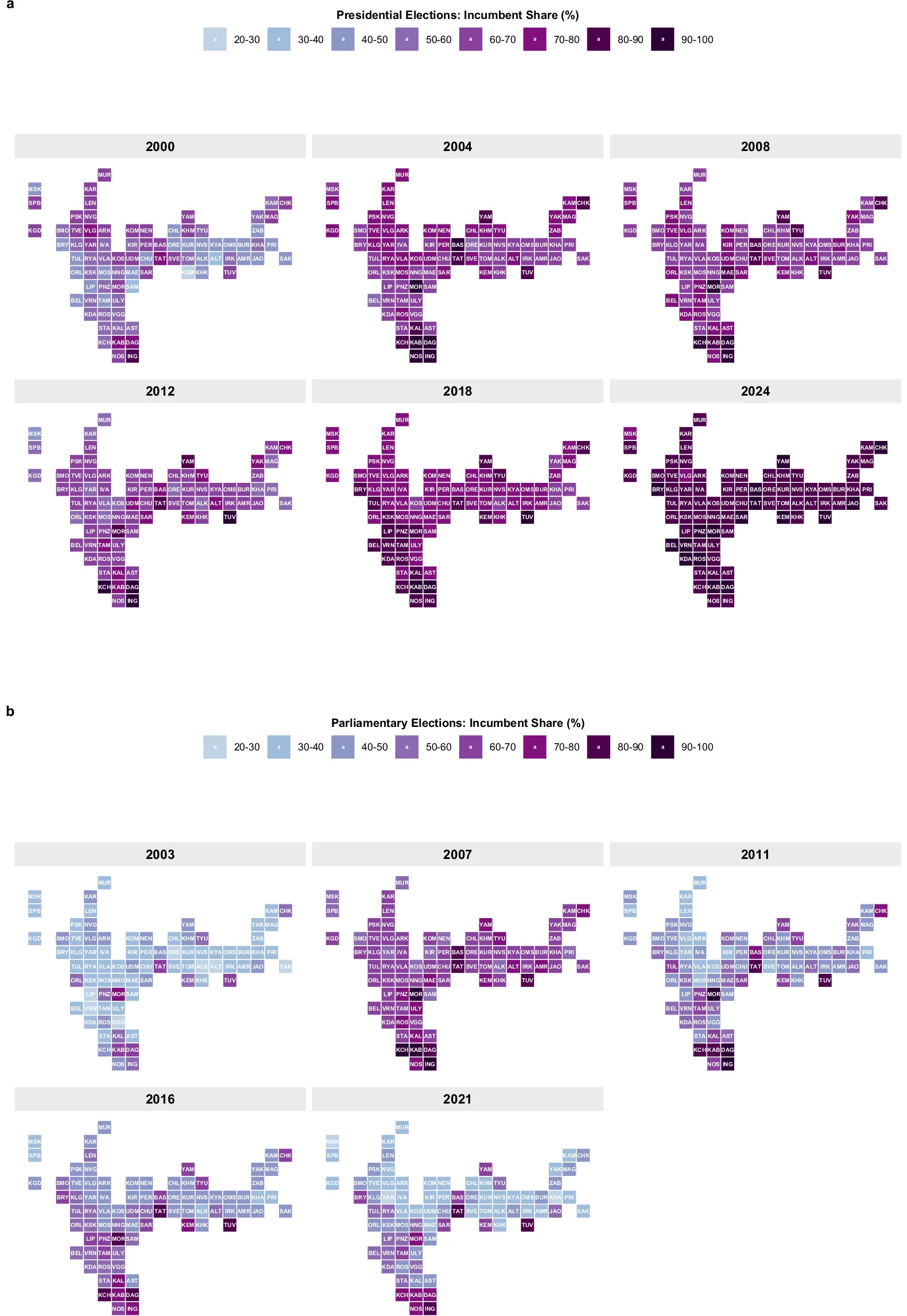
Incumbent support across Russian regions. **Abbreviations of Russian Regions used in the text:** ADY - Respublika Adygeya; ALT - Respublika Altay; ALK - Altayskiy kray; AMR - Amurskaya oblast’; ARK - Arkhangel’skaya oblast’; AST - Astrakhanskaya oblast’; BAS - Respublika Bashkortostan; BEL - Belgorodskaya oblast’; BRY - Bryanskaya oblast’; BUR - Respublika Buryatiya; BYK - Gorod Baykonur; CHE - Chechenskaya Respublika; CHK - Chukotskiy avtonomnyy okrug; CHL - Chelyabinskaya oblast’; CHU - Chuvashskaya Respublika - Chuvashiya; DAG - Respublika Dagestan; ING - Respublika Ingushetiya; IRK - Irkutskaya oblast’; IVA - Ivanovskaya oblast’; JAO - Yevreyskaya avtonomnaya oblast’; KAB - Kabardino-Balkarskaya Respublika; KAL - Respublika Kalmykiya; KAM - Kamchatskiy kray; KAR - Respublika Kareliya; KCH - Karachayevo-Cherkesskaya Respublika; KDA - Krasnodarskiy kray; KEM - Kemerovskaya oblast’ - Kuzbass; KGD - Kaliningradskaya oblast’; KHA - Khabarovskiy kray; KHK - Respublika Khakasiya; KHM - Khanty-Mansiyskiy avtonomnyy okrug - Yugra; KIR - Kirovskaya oblast’; KLG - Kaluzhskaya oblast’; KOM - Respublika Komi; KOS - Kostromskaya oblast’; KSK - Kurskaya oblast’; KUR - Kurganskaya oblast’; LEN - Leningradskaya oblast’; LIP - Lipetskaya oblast’; MAE - Respublika Mariy El; MAG - Magadanskaya oblast’; MOR - Respublika Mordoviya; MOS - Moskovskaya oblast’; MSK - gorod Moskva; MUR - Murmanskaya oblast’; NEN - Nenetskiy avtonomnyy okrug; NNG - Nizhegorodskaya oblast’; NOS - Respublika Severnaya Osetiya - Alaniya; NVG - Novgorodskaya oblast’; NVS - Novosibirskaya oblast’; OMS - Omskaya oblast’; ORE - Orenburgskaya oblast’; ORL - Orlovskaya oblast’; OUT - Territoriya za predelami RF; PEN - Penzenskaya oblast’; PER - Permskiy kray; PRI - Primorskiy kray; PSK - Pskovskaya oblast’; ROS - Rostovskaya oblast’; RYA - Ryazanskaya oblast’; SAK - Sakhalinskaya oblast’; SAM - Samarskaya oblast’; SAR - Saratovskaya oblast’; SMO - Smolenskaya oblast’; SPB - gorod Sankt-Peterburg; STA - Stavropol’skiy kray; SVE - Sverdlovskaya oblast’; TAM - Tambovskaya oblast’; TAT - Respublika Tatarstan (Tatarstan); TOM - Tomskaya oblast’; TUL - Tul’skaya oblast’; TUV - Respublika Tyva; TVE - Tverskaya oblast’; TYU - Tyumenskaya oblast’; UDM - Udmurtskaya Respublika; ULY - Ul’yanovskaya oblast’; VGG - Volgogradskaya oblast’; VLA - Vladimirskaya oblast’; VLG - Vologodskaya oblast’; VRN - Voronezhskaya oblast’; YAK - Respublika Sakha (Yakutiya); YAM - Yamalo-Nenetskiy avtonomnyy okrug; YAR - Yaroslavskaya oblast’; ZAB - Zabaykal’skiy kray.

Using tile grid maps, Fig. 3 visualizes the share of votes received by incumbent parties and candidates in Russian federal elections across regions. Each tile represents a federal subject (region) of Russia, labeled with its abbreviation (see legend for full names). The top panel (a) displays results from parliamentary elections, showing support for the ruling party United Russia (2003–2021). The bottom panel (b) presents presidential election results, covering Vladimir Putin’s campaigns (2000, 2004, 2012, 2018, and 2024) and Dmitry Medvedev’s term (2008). Color shading indicates the percentage of votes received by the incumbent in each region, grouped into 10 percentage-point bins. To enable consistent comparison over time, just as in the precinct-level dataset, regional boundaries were harmonized to reflect the administrative-territorial structure as of 2024. Examining the region-level spatial perspective is important because Russia is a federal state by constitutional design, and regional variation in political dynamics was pronounced in the early post-Soviet period. In the early 2000s, incumbent support varied widely across federal subjects, reflecting diverse local political landscapes and varying degrees of autonomy. Over time, however, this variation has markedly declined with the construction of ‘power vertical’^23,72,74^. The maps illustrate the progressive consolidation of the regime and a de facto centralization of the Russian political system, in which regional differentiation in electoral outcomes has largely faded, giving way to uniform patterns of high support for federal incumbents.

Presidential elections (panel b) reveal dramatic increases in incumbent support, especially in industrial and peripheral regions. Kemerovo oblast’ (Kuzbass) rose from 24.4% in 2000 to 95.5% in 2024, the largest nationwide gain (+71 pp). Jewish AO, Bryansk, and Samara also posted increases of nearly 50 points. By contrast, regions like Karelia, Novgorod, and Yaroslavl’ saw modest gains of just 13–15 points, and Ingushetia—already highly supportive in 2000—barely changed. Chechnya remained an outlier, exceeding 99% in both 2012 and 2024. The only low outlier outside of 2000 was Moscow in 2012 (47.5%), amid large-scale urban protests.

Parliamentary elections (panel a) follow a similar consolidation trend. In 2003, the support of the incumbent party ranged widely - from just 24.2% in the Altay Republic to more than 82.4% in Mordovia. By 2021, United Russia’s dominance had expanded sharply in regions like Ingushetia, Karachay-Cherkessia, and Bryansk (+30–33 pp). However, a handful of regions, including Chukotka (11.3 pp), Khabarovsk (11.1 pp), and Moscow (5.1 pp), recorded slight declines, although support remained high in absolute terms. Together, these patterns illustrate Russia’s shift from early post-Soviet pluralism toward an increasingly centralized and uniform electoral regime, with regional differentiation in outcomes sharply diminished over time.

### Indicators of Electoral Fraud and Voter Intimidation

There is substantial and growing evidence of electoral fraud and voter intimidation in Russian elections, supported by both observational and experimental research designs^17,18,20,73,75,76^. However, much of the existing literature has paid limited attention to the temporal dimension of these processes, largely due to the challenges of compiling consistent data over extended time periods. In this context, we hope the dataset presented here can help fill this gap. While this section illustrates select correlates of electoral fraud and voter coercion, a precise identification and quantification of these phenomena lies beyond the scope of this data descriptor.

We measure incumbent vote share as the proportion of all registered voters (i.e., absolute vote share), rather than as a proportion of valid votes cast (relative vote share). Following Myagkov, Ordeshook and Shakin^75^, this absolute measure is more appropriate for detecting electoral manipulation, as it captures the dual-margin strategy whereby both turnout and vote choice are simultaneously inflated at manipulated precincts.

**Fig. 4:**
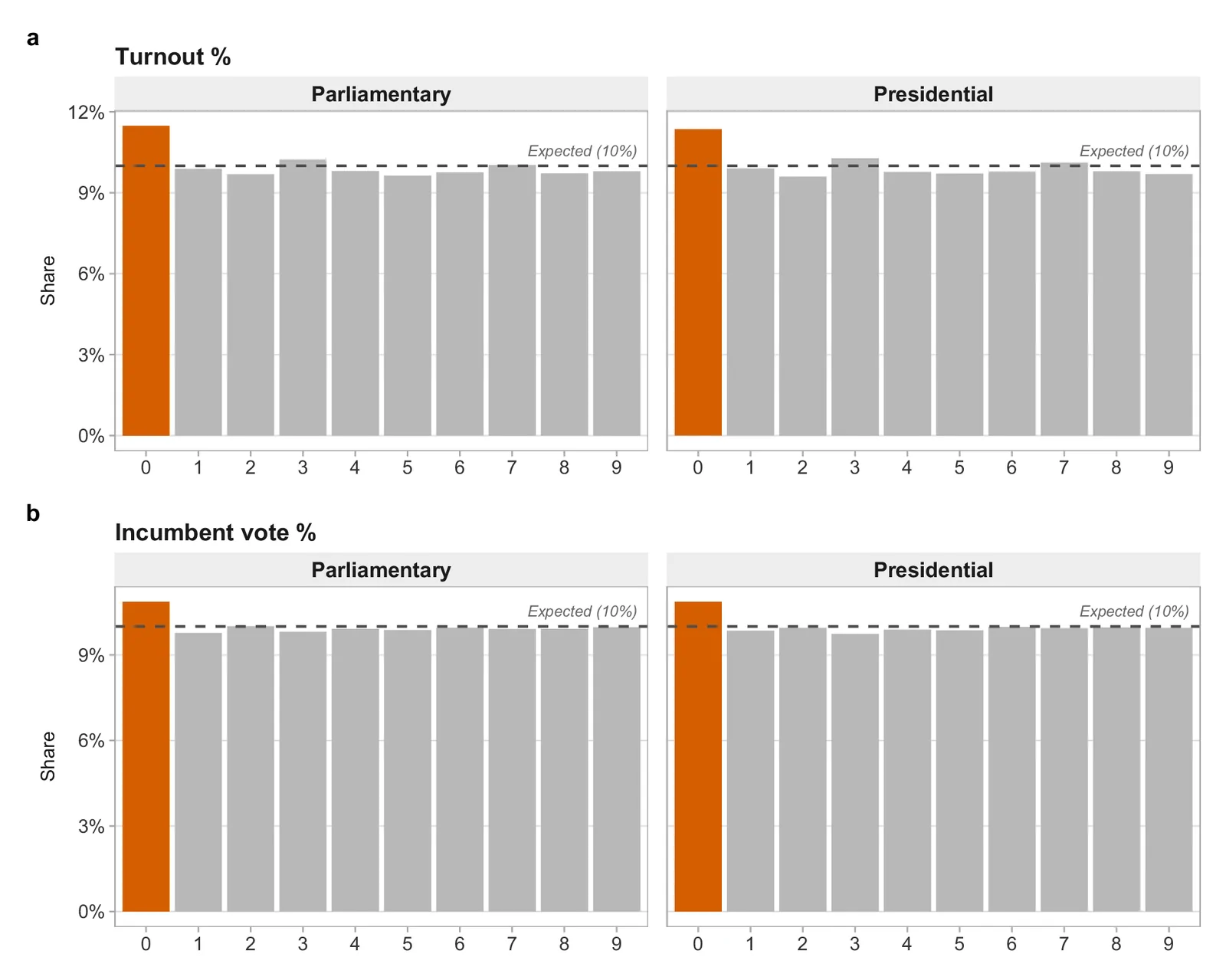
Distribution of last-digit frequencies (0–9) in turnout percentages (top row) vs. absolute incumbent vote percentages (bottom row) for parliamentary (left) and presidential (right) elections, 2000–2024. The dashed line at 10% represents the expected frequency for each digit under a uniform distribution. Orange bars highlight the overrepresented digit ‘0’.

Figure 4 presents the distribution of the last digit frequencies (0–9) in official turnout percentages (top row) and incumbent vote shares (bottom row) in Russian parliamentary (left column) and presidential (right column) elections from 2000 to 2024. The dashed horizontal line at 10% denotes the expected frequency for each digit under a uniform distribution, assuming natural occurrence data. Deviations from this baseline may indicate human manipulation or reporting bias.

In all four panels, the digit ‘0’ (highlighted in orange) is notably overrepresented, with frequencies exceeding 15% in turnout data and 12% in incumbent results, especially in parliamentary elections. This pattern is inconsistent with the expected random distribution and suggests a systemic rounding or fabrication bias favoring round numbers. Such anomalies are often interpreted as potential indicators of electoral manipulation or artificial result engineering.

In Russia’s authoritarian electoral system, voter mobilization often takes place through workplace-based coercion, where employers – especially those in state-owned or state-dependent firms – serve as intermediaries for the regime. Rather than relying solely on vote buying or overt fraud, authorities use economic pressure, including job threats or service withdrawal, to compel turnout and support for pro-regime candidates^73^. It is shown that such coercion is widespread, particularly in single-company towns, where 44% of workers reported intimidation during the 2011–2012 election cycle^73^. This strategy allows the state to mobilize voters effectively while minimizing the visibility of direct electoral manipulation.

**Fig. 5:**
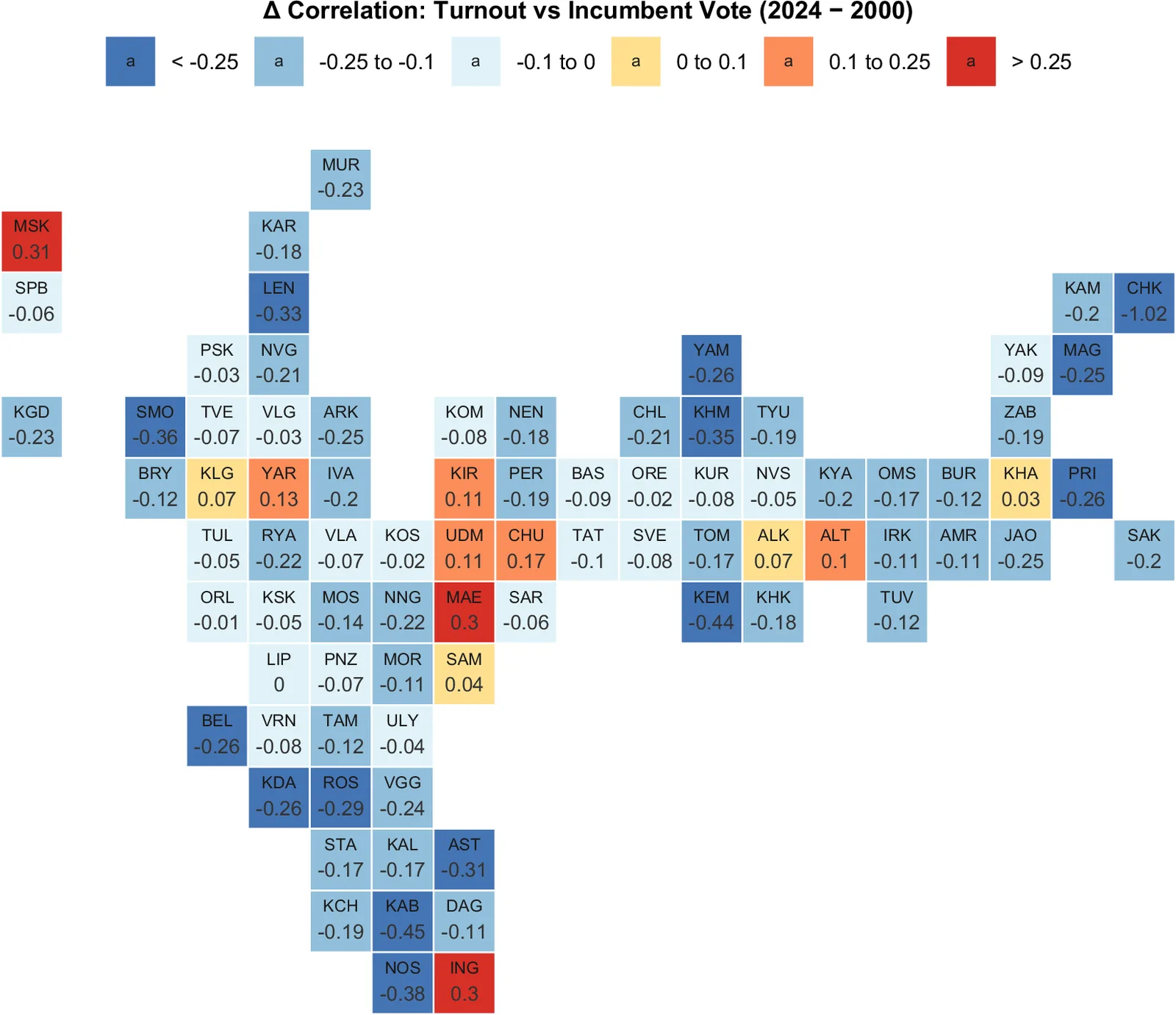
Change in the correlation between turnout and incumbent absolute vote share across Russian regions from 2000 to 2024.

Figure 5 shows the change in the correlation between turnout and incumbent vote share across Russian regions from 2000 to 2024. The map reveals that in most regions (shaded in purple and blue), this correlation has declined over the period. In 2000, many regions showed positive correlations between turnout and incumbent support, consistent with genuine logic of electoral mobilization. By 2024, this relationship has weakened or inverted in most areas, with particularly sharp declines in Chukotka (−1.02), Kabardino-Balkaria (−0.45), and Kemerovo (−0.44). Some regions, notably Moscow (0.31), Ingushetia and Mari El (0.25), show strengthening positive correlations—the classic signature of ballot-stuffing where artificially inflated turnout correlates with disproportionate incumbent support. The widespread negative trend suggests Russian electoral manipulation has evolved beyond crude ballot-stuffing toward more sophisticated methods that do not produce the traditional turnout-fraud correlation, such as selective opposition suppression^13^, manipulation of alternative voting modes^68,69^, or geographically concentrated fraud that operates below the regional aggregation level.

One of the other potential tools of electoral fraud is off-site voting, where transparency monitoring is more difficult. Unofficially known as ‘home voting’, off-site voting involves members of the precinct election commission physically traveling to voters’ private residences, typically homes, apartments, or hospital rooms, with a portable ballot box, election materials, and the local voter list. It is officially reserved for voters who are elderly, sick, disabled, or otherwise unable to reach a polling station. Crucially, this method does not require the creation of a separate or temporary polling place, distinguishing it from other forms of early or mobile voting. The voting takes place in private, non-public settings, making the process inherently opaque and susceptible to undue influence or manipulation. Independent observers are often absent or restricted, and procedural documentation is typically minimal, raising additional concerns about the integrity and verifiability of ballots cast in this manner.

**Fig. 6:**
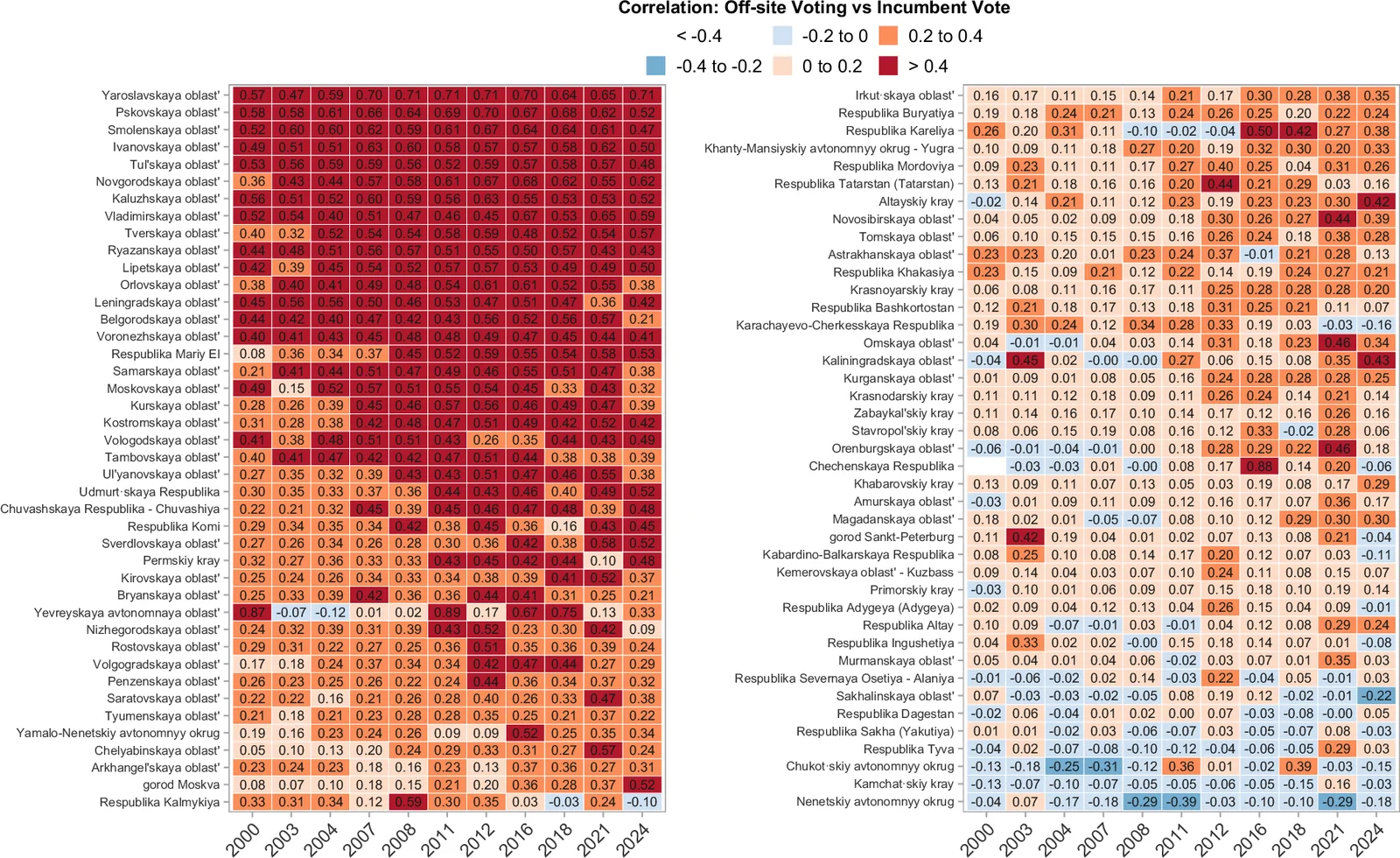
Correlation between off-site voting share and incumbent absolute vote share by region and year.

Figure 6 shows how the correlation between off-site voting and incumbent absolute vote share varies across Russian regions and election years from 2000 to 2024. The heatmap reveals a strongly uneven geography. In many western and central Russian oblasts, the association is consistently positive and often strong, while several peripheral regions and ethnic republics display weak, near-zero, or negative correlations.

The clearest cases of persistent positive association are concentrated in the western and central parts of the country. In *Yaroslavskaya oblast’*, the correlation remains among the highest in the dataset, reaching 0.70 in 2007 and 2016, 0.71 in 2008, 2012, and 2024, and never falling below 0.47. *Pskovskaya oblast’* shows a similarly strong pattern, rising from 0.58 in 2000 to 0.69 in 2011 and 0.70 in 2012, before remaining positive in later elections. Other oblasts, including *Ivanovskaya*, *Tulskaya*, *Kaluzhskaya*, *Vladimirskaya*, *Ryazanskaya*, and *Lipetskaya*, also frequently record correlations above 0.50. These cases suggest a durable linkage between off-site voting and higher incumbent results in a substantial cluster of regions.

The figure also highlights regions where the relationship strengthens over time. *Kaliningradskaya oblast’* moves from a near-zero or negative correlation in 2000–2008 to 0.35 in 2021 and 0.43 in 2024. *Novosibirskaya oblast’* similarly rises from very low correlations in the early 2000s to 0.44 in 2021 and 0.39 in 2024. *Irkutskaya oblast’* increases from 0.16 in 2000 to 0.38 in 2021 and 0.35 in 2024, while *Respublika Kareliya* shifts from moderate values in the 2000s to 0.50 in 2016 and remains positive afterward. These cases are more suggestive of a growing association than regions where the correlation is merely stable at a moderate level.

At the same time, the pattern is not uniform across Russia. Some regions exhibit weak, unstable, or negative correlations. In the *Chechen Republic*, the correlation remains close to zero or negative in most election years, including −0.03 in 2000, −0.03 in 2003, and −0.06 in 2024. *Respublika Sakha (Yakutiya)* also displays consistently weak relationships, hovering around zero across most years and reaching −0.03 in 2024. *Respublika Tyva* shows mostly negative or near-zero correlations for much of the period, despite a temporary increase in 2021. Far Eastern and northern regions show particularly unstable or negative patterns: *Kamchatskiy kray* shifts from positive correlations in the 2000s to −0.15 in 2016 and −0.31 in 2024, while *Chukotskiy avtonomnyy okrug* and *Nenetskiy avtonomnyy okrug* also display several negative values.

Overall, the heatmap suggests that off-site voting is not associated with incumbent support in the same way everywhere. The strongest and most persistent positive correlations appear in western and central oblasts, whereas weak, negative, or unstable correlations are more common in parts of the Far East, the North, the North Caucasus, and several ethnic republics. This heterogeneity cautions against treating off-site voting as a uniform mechanism of electoral manipulation across Russia. Instead, its relationship with incumbent support appears to be regionally embedded and likely shaped by local administrative practices, monitoring conditions, and the broader repertoire of electoral control.

Together, these patterns underscore regional heterogeneity in electoral control strategies: while some areas increasingly rely on off-site voting to amplify incumbent support, others maintain uniformly high results through other means—whether through direct ballot manipulation, complete administrative control that renders off-site voting redundant, or genuinely different electoral dynamics in remote regions. The growing strength and consistency of these correlations across dozens of federal subjects in European Russia suggest that off-site voting is not merely a logistical option but an increasingly central mechanism in Russia’s authoritarian electoral toolkit, particularly in regions where more sophisticated administrative mobilization strategies have been developed.

## Technical Validation

To validate the technical quality of our dataset, we benchmarked key aggregate indicators against official election statistics published by the Central Election Commission of the Russian Federation (CEC RF). Specifically, we compared the ratio of officially reported turnout and number of registered voters across all federal elections from 2000 to 2024. As shown in Table 5, the RED electorate totals closely track official figures in most years, while turnout totals show larger divergences in elections where excluded territories or non-precinct voting modes account for a greater share of official results. The largest gap appears in 2024, when RED captures 88.58% of official turnout but 98.21% of the official electorate, reflecting the exclusion of internationally non-recognized territories and the aggregation/reporting structure of non-precinct voting. This benchmarking supports the reliability and internal consistency of the compiled dataset.

**Table 5:** Election data comparison: database vs. official reported totals.

Year	Election Type	Off. Turnout	Off. Voters	RED Turnout	RED Voters	Prec. Turnout	Prec. Voters
2000	Presidential Election	75,070,776	109,372,046	73,275,108	106,293,097	97.61%	97.18%
2003	Parliamentary Election	60,712,301	109,373,000	60,763,612	109,001,826	100.08%	99.66%
2004	Presidential Election	69,504,278	108,064,281	69,368,305	107,848,174	99.80%	99.80%
2007	Parliamentary Election	69,537,065	109,145,517	69,405,157	108,882,656	99.81%	99.76%
2008	Presidential Election	74,717,992	107,222,016	74,630,484	106,970,974	99.88%	99.77%
2011	Parliamentary Election	65,722,412	109,820,679	65,579,252	109,096,368	99.78%	99.34%
2012	Presidential Election	71,701,665	109,860,331	71,475,138	109,604,972	99.68%	99.77%
2016	Parliamentary Election	53,646,688	111,724,534	52,378,595	109,544,484	97.64%	98.05%
2018	Presidential Election	73,578,992	109,008,428	72,839,536	108,175,669	99.00%	99.24%
2021	Parliamentary Election	56,483,685	109,204,662	53,410,571	108,088,833	94.56%	98.98%
2024	Presidential Election	82,985,310	108,091,103	73,504,820	106,151,970	88.58%	98.21%

**Notes:** The divergence between database and official reported results is mainly due to: (1) In 2000, the absence of the data from Chechnya as well as parts of Altai Krai, Kaliningrad Oblast, Omsk Oblast and Dagestan; (2) From 2016, the exclusion of results from internationally non-recognized federal subjects, namely the Autonomous Republic of Crimea and Sevastopol; (3) In 2024, the additional exclusion of non-recognized subjects: Donetsk and Luhansk People’s Republics, Zaporizhia, and Kherson Oblasts.

## Usage Notes

The RED dataset^21^ enables fine-grained analyses of electoral dynamics in Russia across six presidential and five parliamentary elections between 2000 and 2024. Given variations in precinct boundaries, commission names, and electoral system design over time, users should exercise caution when performing longitudinal analyses and rely on the provided harmonized identifiers for consistency. Data for certain regions in 2000 (e.g., Chechnya) may be incomplete. Online voting results are provided at the electoral district level for 2021 and regional level for 2024 to support analysis of aggregate trends and to clarify which territories had voting activity not fully captured by the precinct-level data.

Precinct (UIK) identifiers are standardized based on the most recent electoral cycle to ensure comparability across elections. These standardized precinct codes may not reflect actual precinct numbers used in earlier election years and may change in future cycles as electoral geography continues to evolve. Users conducting panel or longitudinal analyses should rely on these harmonized identifiers rather than original precinct codes. Note that while region mergers, redistricting, municipal splits, mergers, territorial transfers and renaming as well as precinct renumbering are addressed, precinct redrawing cases are not captured, which may introduce measurement error in panel analyses.

## Data Availability

The dataset^21^ and codebook are available at Harvard Dataverse https://doi.org/10.7910/DVN/DFGNTP in a comma-separated format compatible with various statistical software. The dataset may be updated in the future to address limitations or incorporate additional data. Users who identify issues, errors, or have suggestions for improvement are invited to contact the authors to support the continued development of RED.

## Code availability

The code used to construct the dataset is available in the RED replication package on GitHub (https://github.com/georgytarasenko/RED-replication-package). All preprocessing and harmonization procedures are fully documented in the replication package.
